# 4-(2,4,6-Trimethyl­benz­yl)-1,3-thia­zol-2-amine

**DOI:** 10.1107/S1600536811006386

**Published:** 2011-02-26

**Authors:** Abel M. Maharramov, Ali N. Khalilov, Atash V. Gurbanov, Mirze A. Allahverdiyev, Seik Weng Ng

**Affiliations:** a Department of Organic Chemistry, Baku State University, Baku, Azerbaijan; b Department of Chemistry, University of Malaya, 50603 Kuala Lumpur, Malaysia

## Abstract

The methyl­ene C atom in the title compound, C_13_H_16_N_2_S, is connected to a five-membered thia­zole ring and a mesityl substituent. The rings are aligned at 75.4 (1)°. The amino substitutent inter­acts with the ring N atom of an adjacent mol­ecule by an inter­molecular N—H⋯N hydrogen bond, generating a helical chain running along the *b* axis.

## Related literature

For background to the synthetic procedure,: see: Yadigarov *et al.* (2010 ▶).
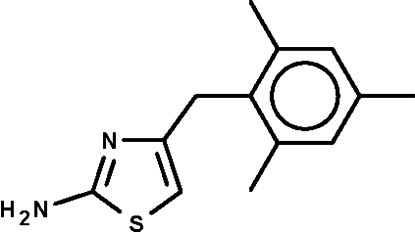


## Experimental

### 

#### Crystal data

C_13_H_16_N_2_S
*M* *_r_* = 232.34
Monoclinic, 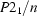

*a* = 5.5028 (5) Å
*b* = 30.832 (3) Å
*c* = 7.8355 (7) Å
β = 110.016 (1)°
*V* = 1249.08 (19) Å^3^
*Z* = 4
Mo *K*α radiation
μ = 0.23 mm^−1^
*T* = 100 K
0.30 × 0.20 × 0.20 mm

#### Data collection

Bruker APEXII diffractometer
Absorption correction: multi-scan (*SADABS*; Sheldrick, 1996 ▶) *T*_min_ = 0.933, *T*_max_ = 0.955
7129 measured reflections
2749 independent reflections
2486 reflections with *I* > 2σ(*I*)
*R*_int_ = 0.024

#### Refinement

*R*[*F* ^2^ > 2σ(*F* ^2^)] = 0.045
*wR*(*F* ^2^) = 0.120
*S* = 1.06
2749 reflections
156 parameters
2 restraints
H atoms treated by a mixture of independent and constrained refinement
Δρ_max_ = 0.38 e Å^−3^
Δρ_min_ = −0.24 e Å^−3^

### 

Data collection: *APEX2* (Bruker, 2005 ▶); cell refinement: *SAINT* (Bruker, 2005 ▶); data reduction: *SAINT*; program(s) used to solve structure: *SHELXS97* (Sheldrick, 2008 ▶); program(s) used to refine structure: *SHELXL97* (Sheldrick, 2008 ▶); molecular graphics: *X-SEED* (Barbour, 2001 ▶); software used to prepare material for publication: *publCIF* (Westrip, 2010 ▶).

## Supplementary Material

Crystal structure: contains datablocks global, I. DOI: 10.1107/S1600536811006386/im2269sup1.cif

Structure factors: contains datablocks I. DOI: 10.1107/S1600536811006386/im2269Isup2.hkl

Additional supplementary materials:  crystallographic information; 3D view; checkCIF report

## Figures and Tables

**Table 1:** Hydrogen-bond geometry (Å, °)

*D*—H⋯*A*	*D*—H	H⋯*A*	*D*⋯*A*	*D*—H⋯*A*
N1—H11⋯N2^i^	0.88 (1)	2.06 (1)	2.907 (2)	163 (2)

Symmetry code: (i) 
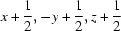
.

**Fig. 1.**
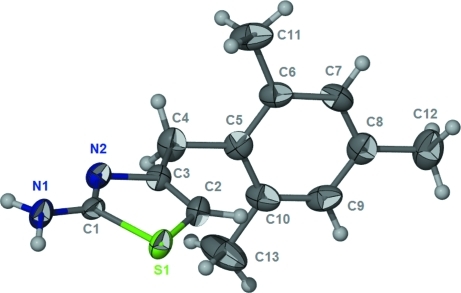
Thermal ellipsoid plot (Barbour, 2001) of C13H16N2S at the 70% probability level; hydrogen atoms are drawn as spheres of arbitrary radius.

## supplementary crystallographic information

### Comment

A recent study reported the reaction of 1-chloro-3-(2,4,6-trimethylphenyl)-propan-2-one with primary amines. The chlorine atom in the α-chloro ketone is not replaced directly by an amino RNH– group. The intermediate product undergoes a Favorskii rearrangement that furnishes a compound having two methylene groups between the aromatic system and the amido unit (Yadigarov *et al.*, 2010). The present study employs thiourea as the amine. One of its amino –NH_2_ groups is involved in the formation of the thiazolyl ring in the resulting product (Scheme I, Fig. 1). The methylene carbon is connected to the five-membered thiazolyl ring and the six-membered mesityl group. The rings are aligned at 75.4 (1) °. The amino –NH_2_ substitutent interacts with the ring N atom of an adjacent molecule by an N–H···N hydrogen bond generating a helical chain that runs along the *b*-axis of the monoclinic unit cell.

### Experimental

1-Chloro-3-(2,4,6-trimethylphenyl)-propan-2-one (10 mmol) and thiourea (10 mmol) were stirred in water (100 ml) for an hour. A precipitate formed and this was collected and redissolved in hot ethanol. Slow evaporation of the solvent gave colorless crystals in 50% yield; m.p. 380–381 K.

### Refinement

Carbon-bound H-atoms were placed in calculated positions [C–H 0.93 to 0.97 Å] and were included in the refinement in the riding model approximation, with *U_iso_*(H) set to 1.2-1.5 *U_eq_*(C).

The amino H-atoms were located in a difference Fourier map and were refined with a distance restraint of N–H 0.88±0.01 Å; their temperature factors were refined isotropically.

### Crystal data

**Table:**

C_13_H_16_N_2_S	*F*(000) = 496
*M**_r_* = 232.34	*D*_x_ = 1.235 Mg m^−^^3^
Monoclinic, *P*2_1_/*n*	Mo *K*α radiation, λ = 0.71073 Å
Hall symbol: -P 2yn	Cell parameters from 3598 reflections
*a* = 5.5028 (5) Å	θ = 2.6–29.1°
*b* = 30.832 (3) Å	µ = 0.23 mm^−^^1^
*c* = 7.8355 (7) Å	*T* = 100 K
β = 110.016 (1)°	Prism, colorless
*V* = 1249.08 (19) Å^3^	0.30 × 0.20 × 0.20 mm
*Z* = 4	

### Data collection

**Table:**

Bruker APEXII diffractometer	2749 independent reflections
Radiation source: fine-focus sealed tube	2486 reflections with *I* > 2σ(*I*)
graphite	*R*_int_ = 0.024
φ and ω scans	θ_max_ = 27.5°, θ_min_ = 2.6°
Absorption correction: multi-scan (*SADABS*; Sheldrick, 1996)	*h* = −7→7
*T*_min_ = 0.933, *T*_max_ = 0.955	*k* = −40→24
7129 measured reflections	*l* = −9→10

### Refinement

**Table:**

Refinement on *F*^2^	Primary atom site location: structure-invariant direct methods
Least-squares matrix: full	Secondary atom site location: difference Fourier map
*R*[*F*^2^ > 2σ(*F*^2^)] = 0.045	Hydrogen site location: inferred from neighbouring sites
*wR*(*F*^2^) = 0.120	H atoms treated by a mixture of independent and constrained refinement
*S* = 1.06	*w* = 1/[σ^2^(*F*_o_^2^) + (0.060*P*)^2^ + 0.6878*P*] where *P* = (*F*_o_^2^ + 2*F*_c_^2^)/3
2749 reflections	(Δ/σ)_max_ = 0.001
156 parameters	Δρ_max_ = 0.38 e Å^−^^3^
2 restraints	Δρ_min_ = −0.24 e Å^−^^3^

### Fractional atomic coordinates and isotropic or equivalent isotropic displacement parameters (Å^2^)

**Table:**

	*x*	*y*	*z*	*U*_iso_*/*U*_eq_	
S1	0.65720 (8)	0.220399 (14)	0.75764 (6)	0.02680 (15)	
N1	0.2450 (3)	0.26387 (5)	0.7802 (2)	0.0283 (3)	
H11	0.351 (3)	0.2757 (6)	0.8795 (19)	0.030 (5)*	
H12	0.080 (2)	0.2637 (8)	0.764 (3)	0.038 (6)*	
N2	0.1780 (3)	0.20379 (5)	0.58369 (19)	0.0241 (3)	
C1	0.3278 (3)	0.23024 (5)	0.7040 (2)	0.0215 (3)	
C2	0.5821 (3)	0.17807 (6)	0.6029 (2)	0.0267 (4)	
H2	0.7056	0.1604	0.5759	0.032*	
C3	0.3239 (3)	0.17382 (5)	0.5263 (2)	0.0244 (3)	
C4	0.1797 (4)	0.13975 (6)	0.3931 (3)	0.0338 (4)	
H4A	0.0544	0.1542	0.2862	0.041*	
H4B	0.0800	0.1217	0.4502	0.041*	
C5	0.3520 (3)	0.11046 (6)	0.3288 (2)	0.0282 (4)	
C6	0.3814 (4)	0.11748 (6)	0.1605 (2)	0.0301 (4)	
C7	0.5436 (4)	0.09018 (7)	0.1069 (3)	0.0344 (4)	
H7	0.5633	0.0950	−0.0075	0.041*	
C8	0.6772 (4)	0.05629 (6)	0.2137 (3)	0.0344 (4)	
C9	0.6454 (4)	0.04995 (6)	0.3799 (3)	0.0365 (4)	
H9	0.7352	0.0269	0.4557	0.044*	
C10	0.4858 (4)	0.07644 (6)	0.4388 (3)	0.0336 (4)	
C11	0.2363 (5)	0.15279 (7)	0.0330 (3)	0.0473 (6)	
H11A	0.2694	0.1807	0.0966	0.071*	
H11B	0.0505	0.1465	−0.0085	0.071*	
H11C	0.2946	0.1541	−0.0719	0.071*	
C12	0.8478 (4)	0.02682 (8)	0.1499 (3)	0.0487 (6)	
H12A	0.9348	0.0438	0.0820	0.073*	
H12B	0.7419	0.0042	0.0711	0.073*	
H12C	0.9776	0.0133	0.2552	0.073*	
C13	0.4571 (7)	0.06770 (8)	0.6208 (3)	0.0609 (8)	
H13A	0.5687	0.0434	0.6802	0.091*	
H13B	0.2767	0.0605	0.6029	0.091*	
H13C	0.5074	0.0936	0.6974	0.091*	

### Atomic displacement parameters (Å^2^)

**Table:**

	*U*^11^	*U*^22^	*U*^33^	*U*^12^	*U*^13^	*U*^23^
S1	0.0157 (2)	0.0299 (2)	0.0334 (3)	−0.00014 (16)	0.00646 (16)	−0.00426 (18)
N1	0.0173 (7)	0.0329 (8)	0.0320 (8)	0.0012 (6)	0.0049 (6)	−0.0077 (7)
N2	0.0193 (7)	0.0246 (7)	0.0269 (7)	−0.0006 (6)	0.0061 (5)	−0.0005 (6)
C1	0.0171 (7)	0.0248 (8)	0.0214 (7)	0.0014 (6)	0.0051 (6)	0.0038 (6)
C2	0.0229 (8)	0.0246 (8)	0.0342 (9)	0.0014 (7)	0.0118 (7)	−0.0012 (7)
C3	0.0241 (8)	0.0230 (8)	0.0267 (8)	0.0000 (7)	0.0096 (6)	0.0010 (7)
C4	0.0296 (9)	0.0303 (9)	0.0407 (10)	−0.0038 (8)	0.0110 (8)	−0.0094 (8)
C5	0.0298 (9)	0.0229 (8)	0.0295 (9)	−0.0032 (7)	0.0072 (7)	−0.0051 (7)
C6	0.0343 (9)	0.0249 (9)	0.0255 (9)	−0.0034 (7)	0.0031 (7)	−0.0002 (7)
C7	0.0414 (10)	0.0374 (11)	0.0234 (9)	−0.0047 (9)	0.0098 (8)	−0.0049 (8)
C8	0.0328 (10)	0.0319 (10)	0.0339 (10)	−0.0014 (8)	0.0055 (8)	−0.0129 (8)
C9	0.0458 (11)	0.0245 (9)	0.0292 (9)	0.0056 (8)	0.0000 (8)	−0.0014 (8)
C10	0.0489 (11)	0.0239 (9)	0.0260 (9)	−0.0024 (8)	0.0102 (8)	−0.0025 (7)
C11	0.0621 (14)	0.0328 (11)	0.0353 (11)	0.0052 (10)	0.0016 (10)	0.0071 (9)
C12	0.0402 (12)	0.0481 (13)	0.0552 (14)	0.0031 (10)	0.0127 (10)	−0.0213 (11)
C13	0.118 (2)	0.0327 (12)	0.0400 (13)	0.0024 (13)	0.0379 (15)	0.0050 (10)

### Geometric parameters (Å, °)

**Table:**

S1—C2	1.7323 (18)	C7—C8	1.383 (3)
S1—C1	1.7415 (16)	C7—H7	0.9500
N1—C1	1.351 (2)	C8—C9	1.386 (3)
N1—H11	0.88 (1)	C8—C12	1.509 (3)
N1—H12	0.87 (1)	C9—C10	1.389 (3)
N2—C1	1.304 (2)	C9—H9	0.9500
N2—C3	1.396 (2)	C10—C13	1.512 (3)
C2—C3	1.346 (2)	C11—H11A	0.9800
C2—H2	0.9500	C11—H11B	0.9800
C3—C4	1.502 (2)	C11—H11C	0.9800
C4—C5	1.515 (2)	C12—H12A	0.9800
C4—H4A	0.9900	C12—H12B	0.9800
C4—H4B	0.9900	C12—H12C	0.9800
C5—C10	1.397 (3)	C13—H13A	0.9800
C5—C6	1.399 (3)	C13—H13B	0.9800
C6—C7	1.393 (3)	C13—H13C	0.9800
C6—C11	1.508 (3)		
			
C2—S1—C1	89.03 (8)	C6—C7—H7	118.8
C1—N1—H11	119.3 (14)	C7—C8—C9	117.63 (18)
C1—N1—H12	115.1 (16)	C7—C8—C12	121.1 (2)
H11—N1—H12	118 (2)	C9—C8—C12	121.3 (2)
C1—N2—C3	110.84 (14)	C8—C9—C10	121.79 (18)
N2—C1—N1	125.03 (15)	C8—C9—H9	119.1
N2—C1—S1	114.43 (12)	C10—C9—H9	119.1
N1—C1—S1	120.50 (13)	C9—C10—C5	119.78 (17)
C3—C2—S1	110.36 (13)	C9—C10—C13	119.37 (19)
C3—C2—H2	124.8	C5—C10—C13	120.84 (19)
S1—C2—H2	124.8	C6—C11—H11A	109.5
C2—C3—N2	115.33 (15)	C6—C11—H11B	109.5
C2—C3—C4	127.16 (16)	H11A—C11—H11B	109.5
N2—C3—C4	117.47 (15)	C6—C11—H11C	109.5
C3—C4—C5	113.94 (15)	H11A—C11—H11C	109.5
C3—C4—H4A	108.8	H11B—C11—H11C	109.5
C5—C4—H4A	108.8	C8—C12—H12A	109.5
C3—C4—H4B	108.8	C8—C12—H12B	109.5
C5—C4—H4B	108.8	H12A—C12—H12B	109.5
H4A—C4—H4B	107.7	C8—C12—H12C	109.5
C10—C5—C6	119.39 (17)	H12A—C12—H12C	109.5
C10—C5—C4	120.04 (17)	H12B—C12—H12C	109.5
C6—C5—C4	120.56 (17)	C10—C13—H13A	109.5
C5—C6—C7	118.95 (17)	C10—C13—H13B	109.5
C5—C6—C11	122.00 (18)	H13A—C13—H13B	109.5
C7—C6—C11	119.01 (18)	C10—C13—H13C	109.5
C8—C7—C6	122.45 (18)	H13A—C13—H13C	109.5
C8—C7—H7	118.8	H13B—C13—H13C	109.5

### Hydrogen-bond geometry (Å, °)

**Table:**

*D*—H···*A*	*D*—H	H···*A*	*D*···*A*	*D*—H···*A*
N1—H11···N2^i^	0.88 (1)	2.06 (1)	2.907 (2)	163 (2)

Symmetry codes: (i) *x*+1/2, −*y*+1/2, *z*+1/2.
